# First report on severe septic shock associated with human Parvovirus B19 infection after cardiac surgery

**DOI:** 10.3389/fcimb.2023.1064760

**Published:** 2023-04-05

**Authors:** Chunlin Xiang, Xiaoxiao Wu, Youkang Wei, Tianlong Li, Xuemei Tang, Yi Wang, Xiaoqin Zhang, Xiaobo Huang, Yiping Wang

**Affiliations:** Department of Intensive Care Unit, Sichuan Academy of Medical Sciences and Sichuan Provincial People’s Hospital, Chengdu, China

**Keywords:** human Parvovirus B19, infection, septic shock, cardiac surgery, metagenomic next generation sequencing

## Abstract

**Background:**

Human Parvovirus B19 (PB19) is a single-stranded DNA virus. Septic shock from viremia is rare with PB19; however, this infection can progress to life-threatening conditions. We report the first case of severe septic shock associated with a PB19 infection after cardiac surgery.

**Case Presentation:**

A 50-year-old Chinese woman received elective double metal valve replacement, including the aortic valve and the mitral valve, under cardiopulmonary bypass (CPB) and suffered severe septic shock on postoperative day (PD) 30. Through the detection of PB19-specific nucleic acids in blister fluid and serum samples *via* metagenomic next-generation sequencing (mNGS), positive serum PB19 IgM and no other proven infection, acute PB19 infection was confirmed. After five days of combined treatment, no further fever or abdominal discomfort was noted, and the patient’s circulation gradually became stable without vasoactive medications.

**Conclusion:**

PB19 may be an unrecognized cause of septic shock, rash, fever of unknown origin or multiple systemic signs and symptoms, especially in immunosuppressed and immunocompetent critically ill patients. Investigations for viral aetiology are needed.

## 1 Introduction

PB19 is a single-stranded DNA virus that causes many clinical disorders, of which the most common are erythema infectiosum (EI), aplastic crisis complicating chronic haemolytic anaemia, self-limiting arthritis, and hydrops foetalis, which lack specificity (Meyer, 2003; Landry, 2016). Septic shock from viremia is rare with PB19; however, this infection can progress to life-threatening conditions. In our extensive review of the literature, only six reports of septic shock were associated with PB19. (Ferraz et al., 2005; Bailey, 2006; Kaur et al., 2011; Panicker et al., 2011; García-Salido et al., 2014; Kuriyama et al., 2016)

The pathogenesis of severe septic shock associated with PB19 is not well understood. PB19 targets erythroid progenitors in the bone marrow by binding to glycosphingolipid globoside, leading to large receptor-induced structural changes triggering cell death either by lysis or by apoptosis mediated by the non-structural 1 protein (NS 1) (Rogo et al., 2014). Transactivation of the IL-6 gene by NS1 may represent a common pathway to parvovirus B19-induced tissue damage in many different sites of the body (Mitchell, 2002). The viral particles or the cellular debris from red blood cell destruction could have initiated the systemic inflammatory activation, resulting in sepsis in this case.

We report the first case of severe septic shock associated with PB19 infection after cardiac surgery, which was successfully treated by immunoglobulin. A review of literature on this situation is also performed.

## 2 Case presentation

Our patient is a 50-year-old Chinese woman. She was hospitalized for exertional dyspnoea and easily induced fatigue that she experienced over the 17 years prior to the admission. The patient had a prior diagnosis of rheumatic valvular disease. At admission, the chest X-ray showed cardiac enlargement. Cardiac sonography revealed severe aortic valve disease (stenosis and regurgitation) and mitral stenosis. Therefore, double metal valve replacement, including the aortic valve and the mitral valve, was suggested and performed. After the surgical intervention, a transesophageal echocardiogram revealed no residual valvular event; however, hypotension and a rapid pulse rate were still observed after the operation (lowest blood pressure 88/44 mmHg; heart rate 112 beats per minute). Through the use of vasoactive medications, fluid resuscitation, anti-infectives and other treatments, the patient was transferred to the department of cardiac surgery in good condition on PD 7.

Unfortunately, the patient’s temperature increased to 38.5°C in the evening of PD 30, and she had shaking chills, diarrhoea, a heart rate of 138 per minute, a respiratory rate of 32 per minute, and a blood pressure of 130/62 mmHg with norepinephrine (0.02 µg/kg/min) infusion. An erythematous rash was present over the trunk and extremities. Persistent fever and diarrhoea developed with persistent circulatory failure, despite empiric antibiotic treatment (piperacillin-tazobactam 4.5 g iv q8 h), which was given after blood culture, stool culture and sputum culture sampling.

**Figure 1:**
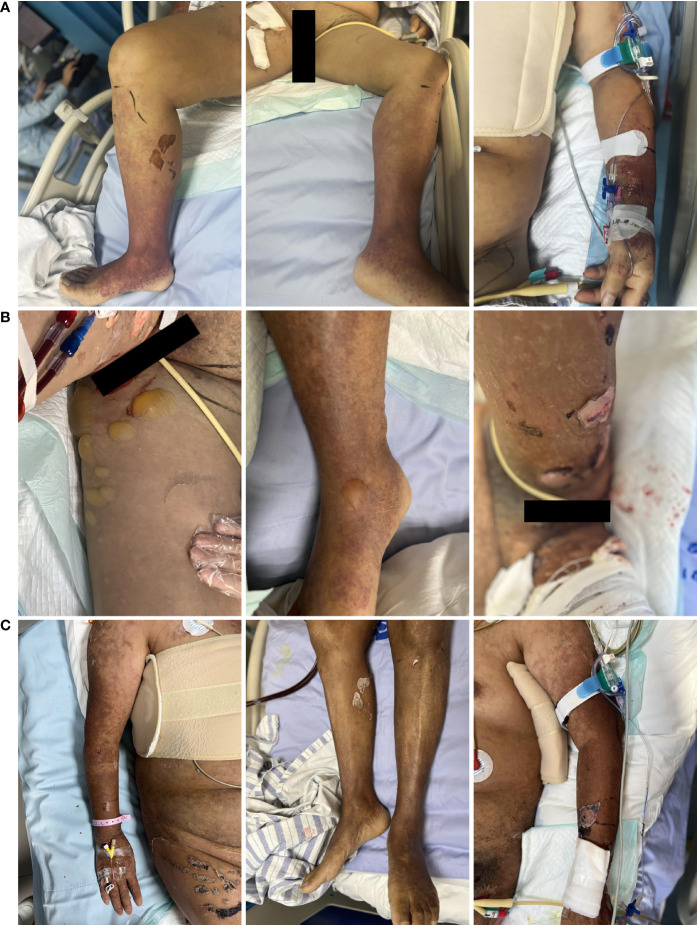
Evolution of the rash. **(A)** The early lesions were erythematous macules or papules approximately 1 to 5 mm in diameter that did not blanch on pressure. The erythematous maculopapular rash was mostly distributed on the arms and lower legs but was less distributed in the trunk. **(B)** The vesicles were pale or yellowish from serous fluid matter. The vesicles slowly become enlarged and ruptured, leaving exuding areas. **(C)** Skin healing with desquamation.

Due to further deterioration of the patient’s circulation, accompanied by continuous fever (up to 41°C), progressive decrease of haemoglobin, anuria and a worsening of the rash on her limbs, the patient was transferred to the SICU for continuous treatment on PD 32. On the physical examination, the blood pressure was 96/70 mmHg with norepinephrine (0.08 µg/kg/min), temperature 37.3°C, respiratory rate of 30 per minute, oxygen saturation 98% with nasal cannula of oxygen-therapy at 4 litres-min, the heart rate was 140 bpm, and a metal sound of the valve was noted at the left-sternal border without murmur, pulmonary crackles, or oedema in the lower extremities. An erythematous maculopapular rash was distributed on the arms and lower legs but did not involve the trunk (**Figure 1A**). There were erythematous macules and papules, approximately 1 to 5 mm in diameter, which did not blanch on pressure. Laboratory data included a white blood cell count of 13.53 × 109/L with 93.3% neutrophils and 4.9% lymphocytes, a platelet count of 174 × 109/L, haemoglobin of 82 g/L, blood lactate of 2.1 mmol/L, hypersensitive C-reactive protein (Hs-CRP) of 228.09 mg/L, procalcitonin (PCT) of 332.00 ng/ml, B-type natriuretic peptide of 71.50 pg/ml, blood glucose of 12.11 mmol/L, and arterial blood lactate of 4.2 mmol/L. Alanine aminotransferase (ALT), aspartate aminotransferase (AST), creatinine (Cr), and blood urea nitrogen (BUN) were variably elevated (**Table 1**). Although the patient had a normal number of B lymphocytes and natural killer cells, the immunologic condition showed decreased counts of CD4+ and CD8+ lymphocytes (CD4+: 135/µl, CD8+: 120/µl).

**Table 1:** The variations trend of laboratory data.

	unit	On admission	PD* 29	PD 30♯	PD 31	PD 32	PD 33	PD 34	PD 35	PD 36	PD 37	PD 38	PD 39	reference range
White blood cell	10^9/L	7.070			9.630	11.610	6.350	11.370	11.620	7.450	8.410	7.660	7.200	3.50-9.50
Neutrophil	10^9/L	5.182			7.271	11.146	5.632	9.994	11.027	6.541	7.207	6.787	5.530	1.80-6.30
Neutrophil ratio	%	73.3			75.5	96.0	88.7	87.9	94.9	87.8	85.7	88.6	76.8	40.0-75.0
Lymphocyte	10^9/L	1.188			1.348	0.302	0.508	0.955	0.256	0.410	0.690	0.421	0.482	1.10-3.20
Hemoglobin	g/L	114			80	79	64	62	70	66	74	77	85	115-150
Platelets	10^9/L	174			343	193	71	56	36	30	30	30	54	101-320
PCT	ng/mL	0.03			0.27	330.40	160.00			43.30			8.74	0-0.05
Hs-CRP	mg/L	6.41			32.36	151.76	208.39	193.01	100.35	120.93	77.89	51.11	29.34	0-5.00
Total bilirubin	umol/l	17.7	11.2		10	11.2	9.9	18.3	25.7	20.2	15.3	13.4	17.1	0.0-21.0
Direct bilirubin	umol/l	5.3	3.6		1.9	8.4	5.8	9.7	15.1	11.5	8.4	8.9	6.6	0.0-8.0
AST	U/L	61	26		30	171	132	133	185	138	64	29	29	13-35
ALT	U/L	106	22		20	57	59	61	79	69	52	37	30	7-40
Cr	umol/l	58.6	78.0		87.0	338.0	320.0	271.0	208.0	211.0	316.0	364.0	374.0	49.0-82.0
BUN	mmol/l	5.55	8.10		7.80	15.10	13.80	13.00	12.10	13.30	31.70	37.70	38.00	2.60-7.50

*PD, postoperative day; ♯ The patient suffered the symptoms of unknown infection at in the evening of PD 30, like fever, shaking chill, diarrhea subsequently.

Bedside transthoracic echocardiography showed that the patient’s right ventricle was in normal size and functioned well, and the patient had enlargement of the left atrium and left ventricle (LA: 50 mm; LAD: 53 mm). The LVEF was 55%, without pericardial effusion. No mechanical prosthetic valve dysfunction was observed. The electrocardiograph showed sinus tachycardia without abnormal ST-T.

The detection of mNGS in the blister fluid and serum from this patient was performed. Empiric antibiotic administration (vancomycin, meropenem and caspofungin) was administered. When the patient’s haemodynamic status gradually worsened, norepinephrine (0.72 µg/kg/min), terlipressin (0.02 µg/kg/min) and cortisol were given due to the emergence of the situation resulting from haemodynamic instability. To decrease the internal milieu disorder in time and clear the various inflammatory mediums efficiently, continuous renal replacement therapy plus hemoperfusion (HA380) was performed. To identify the pathogen as soon as possible, blood culture, stool culture and sputum culture were obtained before antimicrobial administration was continued.

Interestingly, vesicles appeared on PD 33 and were pale or yellowish from serous fluid matter. The vesicles slowly became enlarged and ruptured, leaving exuding areas (**Figure 1B**).

**Figure 2:**
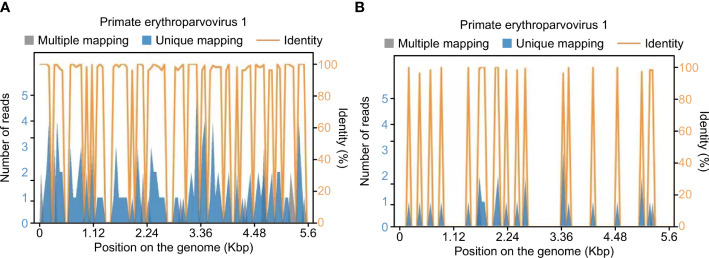
Human Parvovirus B19-specific nucleic acids were detected in mNGS samples of serum and blister fluid. Sequence distribution map: This part only shows the sequence distribution map of species with ≥3 nonrepeat specific sequences detected. **(A)** Serum samples. The total length covered on the genome is 3623 bp, the coverage is 64.7427%, and the average depth is 1.65X. **(B)** Blister fluid samples. The total length covered on the genome is 1059 bp, the coverage is 18.9242%, and the average depth is 1.18X.

After transfer to our department, PB19-specific nucleic acids were detected in mNGS samples of the patient’s blister fluid and serum, and the fluids were negative for other viral, fungal, or bacterial nucleic acids (**Figure 2**). Confirmatory examination showed that PB19 IgM and IgG in serum both were positive (**Table 2**). We checked the antefebrile serum antibody, when PB19 IgM and IgG were both positive after fever. Interestingly, confirmatory examination of antefebrile serum antibody showed that PB19 IgG in serum was positive, while IgM was negative (**Table 2**). All of the cultures, including blood, stool and sputum, obtained negative results. The serology of HIV, HBV, HCV, EBV, CMV, *Toxoplasma gondii* and *Mycoplasma pneumoniae* were all negative. Laboratory investigations revealed a negative serum (1,3)-β-D-glucan test and galactomannan antigen test. The hypocomplementemia complement component 3 concentration was 0.44 [normal 0.9–1.8] g/L; the complement component 4 concentration was 0.08 [normal 0.100–0.400] g/L). Serologic testing showed elevated immunoglobulin E (408.00 [normal 0.00–100.00] IU/mL), but the levels of the other immunoglobulins were normal. The autoimmune antibody profile and pemphigus antibodies were within normal limits. Based on these clinical findings, a multidisciplinary consultation was organized. We substituted vancomycin, meropenem and caspofungin with 4.5 g of intravenous piperacillin-tazobactam every 8 hours. On this basis, this patient was treated with intravenous immunoglobulin (IVIG) 400 mg/kg/d for 5 consecutive days and methylprednisolone 1 mg/kg/d. Topical measures were performed to prevent secondary infections and facilitate the healing of the blisters. After five days of the combined treatment, no further fever or abdominal discomfort was noted, and the patient’s circulation gradually stabilized without vasoactive medications (**Figure 3**) (Gaies et al., 2010). The skin healed with desquamation (**Figure 1C**).

**Table 2:** The changes of IgM and IgG in serum.

	IgM	IgG	Normal range	test method
PD 28#	**-**	**+**	**-**	ELISA
PD 33*	**+**	**+**	**-**	ELISA

# 28 days after operation; *33 days after operation and 4 days after fever.

**Figure 3:**
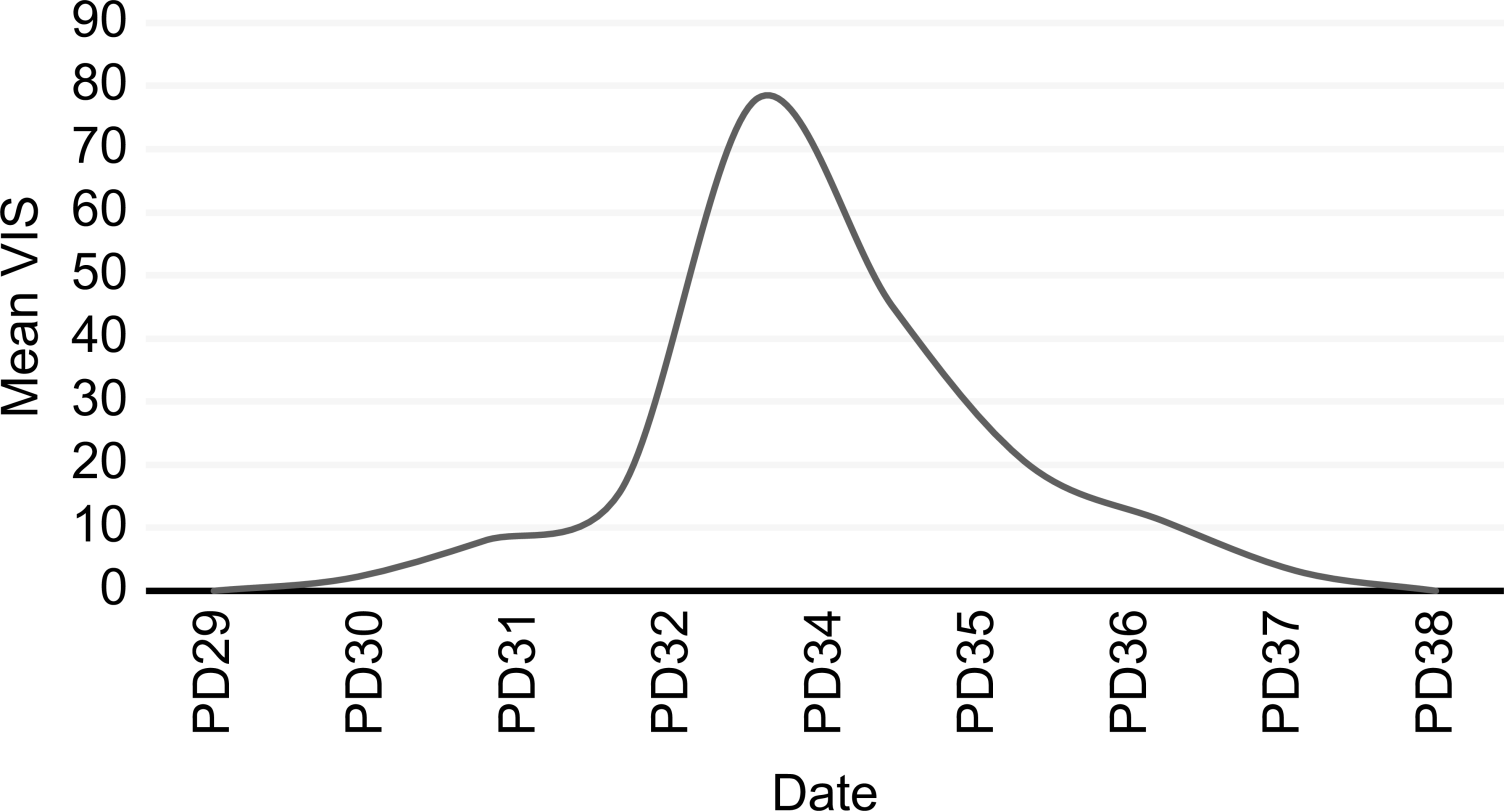
The trend of the mean VIS. #PD, postoperative day; *VIS, Vasoactive–Inotrope Scoring. VIS= dopamine dose (µg/kg/min) + dobutamine dose (µg/kg/min) + 100* epinephrine dose (µg/kg/min) + 10*milrinone dose (µg/kg/min) + 10,000* vasopressin dose (U/kg/min) + 100* norepinephrine dose (µg/kg/min). The hourly doses of the following inotropic and vasoactive medications were recorded every day: dopamine, dobutamine, epinephrine, norepinephrine, milrinone, and vasopressin. The mean VIS was obtained by averaging the hourly scores during 24-hr periods.

## 3 Discussion

PB19 infection is usually a self-limited disease (Landry, 2016). Due to the difficulty in isolating the virus by conventional cell culture, the diagnosis of PB19 relies on serology and DNA detection (Young and Brown, 2004; Landry, 2016). The evidence supporting acute PB19 infection in our case was derived from the presence of PB19-specific nucleic acids (in serum and blister fluid), positive serum PB19 IgM and no other proven infection (Miron et al., 2006; Landry, 2016). Although PB19 acute infections of children can result in EI, up to 50% infection is asymptomatic. A prodromal nonspecific illness consisting of fever, chills, headache, malaise, and myalgias, coinciding with PB19 viremia, can occur, followed by a typical “slapped cheek” facial rash and a lacy, reticular erythematous rash on the trunk and extremities coincident with the immune response. (Landry, 2016) But in adult patients, the typical EI rash is much less common and primary PB19 infection is associated with polymorphous skin manifestations with four predominant, sometimes overlapping, patterns:exanthema, which was reticulated and annular in some cases; the gloves-and-socks pattern; the periflexural pattern; and palpable purpura. Concomitantly, the rash predominated on the legs, trunk, and arms, with a lower frequency of facial involvement. (Drago et al., 2014; Mage et al., 2014). Likewise, at an earlier stage of the disease, the gloves-and-socks pattern and palpable purpura were observed on the lower legs, trunk, and arms without pruritus in our case. And these lesions gradually became erythematous with vesiculation.

Septic shock from viremia rarely occurs with PB19; however, this infection can progress to life-threatening conditions, as seen in our patient. This is the first report on severe septic shock associated with PB19 infection after cardiac surgery. In our extensive review of the literature, only six reports of septic shock were associated with PB19 (Ferraz et al., 2005; Bailey, 2006; Kaur et al., 2011; Panicker et al., 2011; García-Salido et al., 2014; Kuriyama et al., 2016). It is noteworthy that there was only one published case in which the patient had sickle cell disease, which is associated with an increased risk of serious and life-threatening infections (Panicker et al., 2011). Most of the patients reported in the case reports were previously healthy, including 3 adult women and 7-year-olds. In contrast, the reported patient’s immunity level was low at the time of hospitalization after cardiac surgery, which might be due to cardiac surgery injury, CPB, blood loss and malnutrition (Angele and Faist, 2002; Gaudriot et al., 2015; Candela et al., 2021). A previous study showed that the bactericidal activity of neutrophil bactericidal agents against *Staphylococcus aureus* was decreased after cardiac surgery (Mekontso-Dessap et al., 2005). Chalk et al. (2013) have also reported dysfunction of pulmonary macrophages, and patients have strong systemic immune depression after CPB. Moreover, Torrance et al. (2018) reported an association between monocyte dysfunction and major surgery in a study, consisting of 119 participants undergoing the elective gastro-intestinal surgery. As a result, immunodepression increased the susceptibility to infection in surgical patients (Angele and Faist, 2002; Volk, 2002). Findings from the Cardiac Surgical Intensive Care Unit records, which were conducted among 6864 patients after cardiac surgery in 2013-2014, indicate that 4.6% of the patients developed nosocomial infections, including 24 (7.5%) that are consistent with the diagnosis of bloodstream infection (Sahu et al., 2016). Klebsiella, Pseudomonas, Enterobacter, *Escherichia coli*, Acinetobacter, and Staphylococcus were the most frequent pathogens isolated from patients with bloodstream infections (Sahu et al., 2016).

However, the pathogenesis of severe septic shock associated with PB19 is not well understood. PB19 targets erythroid progenitors in the bone marrow by binding to glycosphingolipid globoside, leading to large receptor-induced structural changes triggering cell death either by lysis or by apoptosis mediated by the non-structural 1 protein (NS 1) (Rogo et al., 2014). Transactivation of the IL-6 gene by NS1 may represent a common pathway to parvovirus B19-induced tissue damage in many different sites of the body (Mitchell, 2002).

Despite the patient’s immunocompromised status, it remains unclear how the reported patient became infected. As previously reported in the literature, in compromised hosts, B19 infection can be acquired *via* the respiratory route, from endogenous reactivation, or from blood products (Landry, 2016). No sign revealing cross infections was found in our case, although nosocomial outbreaks of PB19 infection have been reported (Pillay et al., 1992; Seng et al., 1994; Miyamoto et al., 2000; Lui et al., 2001). Strong evidence reveals that PB19 DNA has been detected in bone marrow samples, peripheral blood, synovium, myocardium, and skin for months and even years after the primary infection and the resolution of symptoms of normal hosts (Cassinotti et al., 1993; Kerr et al., 1995; Cassinotti et al., 1997; Cassinotti and Siegl, 2000; Heegaard et al., 2002; Hokynar et al., 2002; Söderlund-Venermo et al., 2002; Schenk et al., 2009). In addition to *de novo* infections, viruses go into latency periods and can be reactivated in both immunosuppressed and immunocompetent critically ill patients (Landry, 2016; Fragkou et al., 2021). The infection in this patient may be explained by secondary reactivation of PB19 due to the patient’s immunocompromised status. The evidence supporting this hypothesis in our case was derived from the positive serum PB19 IgG, while IgM was negative on PD28 (2 days before fever). IgG is a protective antibody, which indicates the past infection. Additionally, the transmission of symptomatic PB19 infection by blood products used during surgery, such as fibrin sealant, has been reported previously (Hino et al., 2000; Enoki et al., 2002; Kawamura et al., 2002; Tournoux et al., 2004). The International Hemovigilance Network collected aggregate data on suspected transfusion-transmitted infections for 2013-2016 from member national hemovigilance systems. In one patient, there was clinical suspicion of PB19 infection obtained by blood transfusion (Politis et al., 2019).

Unfortunately, no antiviral therapy is available to treat PB19 infection (Rogo et al., 2014; Landry, 2016; Fragkou et al., 2021). The treatment of choice mainly includes red-cell transfusion, adjustment in medications to restore or improve the patient’s immune system, and administration of IVIG (Szenborn, 2011; Landry, 2016). A regimen of 400 mg/kg/day for five to ten days has been suggested (Young and Brown, 2004). However, the efficiency of IVIG in severe septic shock patients is unclear. Because the patient’s circulation gradually stabilized with clinical improvements after treatments, we believe that IVIG was beneficial in this case.

In our case, there is a limitation, which should be mentioned. Serum procalcitonin was markedly elevated, and there might be other unrecognized infections, although mNGS, all of the cultures, including blood, stool and sputum, and serology of HIV, HBV, HCV, EBV, CMV, Toxoplasma gondii and Mycoplasma pneumoniae obtained negative results except PB19.

The case presented provides evidence that PB19 may be an unrecognized cause of septic shock, rash, fever of unknown origin or multiple systemic signs and symptoms, especially in immunosuppressed and immunocompetent critically ill patients. Investigations for viral aetiology are needed.

## Data availability statement

The original contributions presented in the study are included in the article/supplementary material. Further inquiries can be directed to the corresponding author.

## Ethics statement

Written informed consent was obtained from the individual for the publication of any potentially identifiable images or data included in this article.

## Author contributions

YPW designed the study. CLX, XXW, and YPW contributed to collection and collation the clinical data. CLX and YKW contributed to extract data from literature and manuscript writing. TLL, XMT, XQZ, XBH and YW contributed to manuscript revisions and approved the final manuscript. All authors contributed to the article and approved the submitted version.
